# Retraction Note: Oscillations of the baseline of solar magnetic field and solar irradiance on a millennial timescale

**DOI:** 10.1038/s41598-020-61020-3

**Published:** 2020-03-04

**Authors:** V. V. Zharkova, S. J. Shepherd, S. I. Zharkov, E. Popova

**Affiliations:** 1 Northumbria University, Department of Mathematics, Physics and Electrical Engineering, Newcastle upon Tyne, NE2 1XE UK; 2 University of Bradford, School of Engineering, Bradford, BD7 1DP UK; 3 University of Hull, Department of Physics and Mathematics, Kingston upon Hull, HU6 7RX UK; 4 Nasir al-Din al-Tusi Shamakhi Astrophysical Observatory Azerbaijan, AZ 1000 Pirqulu, Azerbaijan; 5 National Research University, Higher School of Economics, 101000 Moscow, Russia

Retraction of: *Scientific Reports* 10.1038/s41598-019-45584-3, published online 24 June 2019

The Editors have retracted this Article.

After publication, concerns were raised regarding the interpretation of how the Earth-Sun distance changes over time and that some of the assumptions on which analyses presented in the Article are based are incorrect.

The analyses presented in the section entitled “Effects of SIM on a temperature in the terrestrial hemispheres” are based on the assumption that the orbits of the Earth and the Sun about the Solar System barycenter are uncorrelated, so that the Earth-Sun distance changes by an amount comparable to the Sun-barycenter distance. Post-publication peer review has shown that this assumption is inaccurate because the motions of the Earth and the Sun are primarily due to Jupiter and the other giant planets, which accelerate the Earth and the Sun in nearly the same direction, and thereby generate highly-correlated motions in the Earth and Sun. Current ephemeris calculations [1,2] show that the Earth-Sun distance varies over a timescale of a few centuries by substantially less than the amount reported in this article. As a result the Editors no longer have confidence in the conclusions presented.

S. I. Zharkov agrees with the retraction. V. V. Zharkova, E. Popova, and S. J. Shepherd disagree with the retraction.
